# The Biological Activities of Vitamin D and Its Receptor in Relation to Calcium and Bone Homeostasis, Cancer, Immune and Cardiovascular Systems, Skin Biology, and Oral Health

**DOI:** 10.1155/2018/9276380

**Published:** 2018-05-22

**Authors:** R. A. G. Khammissa, J. Fourie, M. H. Motswaledi, R. Ballyram, J. Lemmer, L. Feller

**Affiliations:** ^1^Department of Periodontology and Oral medicine, Sefako Makgatho Health Sciences University, Medunsa, Pretoria, 0204, South Africa; ^2^Department of Dermatology, Faculty of Health Sciences, Sefako Makgatho Health Sciences University, Medunsa, 0204 Pretoria, South Africa

## Abstract

Vitamin D plays an important role in calcium homeostasis and bone metabolism, with the capacity to modulate innate and adaptive immune function, cardiovascular function, and proliferation and differentiation of both normal and malignant keratinocytes. 1,25(OH)_2_D, the biologically active form of vitamin D, exerts most of its functions through the almost universally distributed nuclear vitamin D receptor (VDR). Upon stimulation by 1,25(OH)_2_D, VDR forms a heterodimer with the retinoid X receptor (RXR). In turn, VDR/RXR binds to DNA sequences termed vitamin D response elements in target genes, regulating gene transcription. In order to exert its biological effects, VDR signalling interacts with other intracellular signalling pathways. In some cases 1,25(OH)_2_D exerts its biological effects without regulating either gene expression or protein synthesis. Although the regulatory role of vitamin D in many biological processes is well documented, there is not enough evidence to support the therapeutic use of vitamin D supplementation in the prevention or treatment of infectious, immunoinflammatory, or hyperproliferative disorders. In this review we highlight the effects of 1,25(OH)_2_D on bone and calcium homeostasis, on cancer, and refer to its effects on the cardiovascular and immune systems.

## 1. Introduction

**Figure 1:**
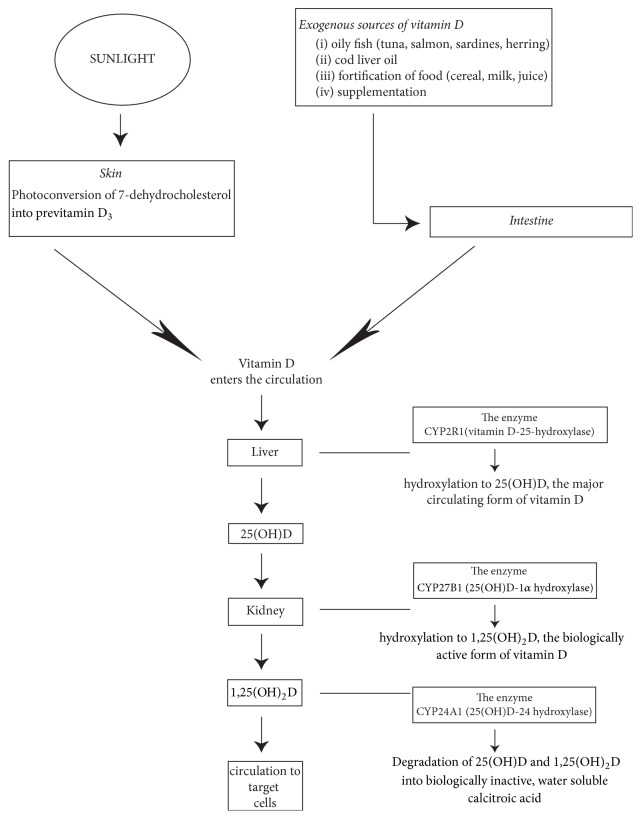
*Synthesis of vitamin D precursors and metabolites.* Photosynthesis in the skin, dietary intake, and supplements are the sources of vitamin D. Melanin and sunblock preparations, which protect the skin from sunlight damage, reduce the UVB penetration resulting in decreased cutaneous photoconversion of 7-dehydrocholesterol to vitamin D [6]. Older persons have a decreased capacity to produce cutaneous previtamin D_3_ [2]. CYP2R1 is the major enzyme responsible for hydroxylation of vitamin D in the liver into 25(OH)D. 25(OH)D is then hydroxylated by the enzyme CYP27B1 in the kidney to become hormonal 1,25(OH)_2_D. CYP27B1 is also expressed by nonrenal tissues [1, 4–6]. 1,25(OH)_2_D, the biologically active form of vitamin D, acts on target cells including cells of the parathyroid glands, osteoblasts, dendritic cells, T cells, and keratinocytes. Small amounts of 1,25(OH)_2_D can also be produced locally in the skin by cutaneous keratinocytes, but only insignificantly contributing to the blood levels of 1,25(OH)_2_D. Usually 25(OH)D and 1,25(OH)_2_D are metabolized by CYP24A1 into water soluble inactive forms which are secreted in bile [7, 8].

Vitamin D and its metabolites are steroid hormones and hormone precursors. About 80% derive from ultraviolet B (UVB) induced photoconversion in the skin of 7-dehydrocholesterol to previtamin D_3_, and the remainder from the diet and from food supplements. Whether derived from skin, food, or supplements, previtamins D_2_ and D_3_ are biologically inactive and, in the liver and in the kidney, undergo two stages of hydroxylation to the biologically active form of vitamin D, 1,25(OH)_2_D (Figure 1). Vitamin D and its metabolites are transported in the circulation by vitamin D binding protein (VDBP), and having reached their target cells, they dissociate from the VDBP and enter the cells [1–8] (Figure 1).

The major function of 1,25(OH)_2_D is to regulate calcium homeostasis [4, 9, 10]; other biological activities include regulation of proliferation and differentiation of several cell lines including keratinocytes, endothelial cells, osteoblasts, and lymphocytes. Most of these biological functions are mediated via the vitamin D nuclear receptor (VDR) which acts as a transcription factor regulating transcription of target genes [1, 4, 10, 11].

Serum levels of 25(OH)D, the major circulating form of vitamin D, are used for screening for vitamin D deficiency in healthy persons, and its normal range is considered to be 75–125 nmol/L [12]. The serum level between 75 and 80 nmol/L of 25(OH)D is generally considered to be optimal for control of circulating parathyroid hormone (PTH) and for maximising calcium absorption and bone mineral density [13].

A serum level of less than <50 nmol/L of 25(OH)D is generally regarded as vitamin D deficiency [14]. The physiological levels of vitamin D may vary: supplementation may be required by the debilitated, the elderly, and those with insufficient exposure to sunlight [5, 6, 13]. The serum level of biologically active 1,25(OH)_2_D depends on multiple factors including the efficacy of synthesis of vitamin D precursor in the skin, on its absorption from dietary sources, on the conversion of 25(OH)D to 1,25(OH)_2_D, and on its transport and its degradation [5, 11]. Common causes of vitamin D deficiency and functional impairment are listed in Table 1. 25(OH)D and 1,25(OH)_2_D are metabolized by CYP24A1 (25(OH)D-24 hydroxylase) into water soluble inactive agents which are secreted in the bile [7, 8] (Figure 1).

Older or obese persons, those with osteoporosis, sarcoidosis, malabsorption syndrome, chronic renal, or hepatic disease, and those taking certain medications (anticonvulsants, systemic glucocorticoids, ketoconazole, or antiretrovirals) are at risk of vitamin D deficiency (Table 1). It appears that supplementation with either vitamin D_2_ or D_3_ is equally effective in correcting vitamin D deficiency [2].

Information from observational clinical studies of the association between vitamin D serum levels and incidence of any diseases is difficult to interpret. Study populations are often heterogeneous, follow-up periods are short, and risk factors such as older age, obesity, smoking, and reduced physical activity are common both to subjects with low 25(OH)D and to subjects with one or other disease. Furthermore, a low 25(OH)D may be a consequence rather than a cause of a disease in people under observation [4].

In this article, we highlight the role of vitamin D and its receptor in regulating the expression of genes involved in calcium homeostasis and bone metabolism, in the function of the immune and the cardiovascular systems, and in modulation of proliferation, differentiation, and apoptosis of keratinocytes.

## 2. Some Genomic Functions of the Vitamin D Receptor (VDR)

The VDR is a nuclear receptor that functions as a transcription factor, and most of its activities are regulated by its ligand, 1,25(OH)_2_D. VDR belongs to the family of steroid receptors which include the adrenal steroid, sex hormone, thyroid hormone, and retinoic acid receptors, and VDR is widely distributed among different cell lineages. However, some of the VDR functions are independent of 1,25(OH)_2_D [4, 15].

VDR forms a heterodimer with the retinoid X receptor (RXR), and in turn VDR/RXR heterodimers bind to DNA sequences in target genes termed vitamin D response elements (VRDEs), resulting in either activation or repression of transcription of target genes [4, 15]. The VDR/RXR-induced transcription of target genes is modulated by other transcriptional coactivators and corepressors, which are recruited to the VDREs [4]. For example, in the epithelium, the DRIP complex of coactivators preferentially binds the VDR of undifferentiated keratinocytes, regulating 1,25(OH)_2_D-induced cell proliferation and expression of early-stage differentiation markers; and the SRC complex of coactivators binds to the VDR in keratinocytes that have already undergone early stages of differentiation and in which the levels of DRIP have declined, inducing the expression of more advanced markers of differentiation [15]. Thus, the interactions of VDR with other transcription coregulators mediate the expression of target genes in keratinocytes in response to ligand-dependent stimulation and also influence ligand-independent cellular functions [16].

In activated T lymphocytes, 1,25(OH)_2_D/VDR/RXR blocks the nuclear factor of activated T cells (NFAT) and the activator protein 1 (AP-1), resulting in repressed transcription of the gene encoding interleukin 2 (lL-2), which is necessary for T cell proliferation and function [1]. 1,25(OH)_2_D also has the capacity to repress the transcription of the key immunological agent interferon gamma (IFN-*γ*), via the binding of the VDR/RXR to a silencer region in IFN-*γ* gene promoter. INF-*γ* is essential for the function of innate and adaptive immunity and its dysregulation is associated with several autoimmunoinflammatory diseases [4, 17]. Similarly, 1,25(OH)_2_D/VDR suppresses the production of IL-17, a cytokine produced by Th17 and which plays a role in the pathogenesis of inflammatory and autoimmune conditions. The repression of transcription of IL-17 by 1,25(OH)_2_D/VDR involves blockade of NFAT, sequestration of the Runt-related transcription factor 1 (Runx 1), and induction of the transcription factor, Foxp3. Foxp3 is also involved in the development and functional activity of regulatory T lymphocytes (Tregs) [4, 18].

VDRs influence several signalling pathways including those regulating bone and calcium homeostasis, inflammation, cell mediated immunity, cell cycle progression, and apoptosis [15]. It has been shown that VDR polymorphism is associated with a risk of cancer, with some genetic variants increasing while others decrease the risk. The VDR-related cancer risk appears to be tissue-specific as it varies in different cancers. The variation between tissues with regard to DNA sequences, to cell phosphorylation, and to coactivators/corepressors may explain why the same VDR variant exerts different biological responses in different tissues [19]. In addition, some genetic variants of VDR may be less responsive to 1,25(OH)_2_D in suppressing inflammatory processes, thus favouring the development of cutaneous inflammatory conditions [20].

Cross sectional studies have shown that chronic use of glucocorticoids is associated with low levels of 25(OH)D [21]. It appears that glucocorticoids can upregulate the renal expression of CYP24A1 which in turn catabolizes 25(OH)D and 1,25(OH)_2_D to water soluble inactive agents thus mediating deficiency of vitamin D [21, 22]. Another link between the use of glucocorticoids and vitamin D is that 1,25(OH)_2_D/VDR and glucocorticoids/glucocorticoid receptor (GR) intracellular signalling pathways cross talk so that increased levels of vitamin D may enhance responsiveness of certain target cells to glucocorticoids [23–25]. Glucocorticoids with their cognate receptors translocate from the cell cytoplasm to the nucleus where they bind to glucocorticoid response element (GRE) to regulate gene transcription. As VDR and GR share some coactivators, VDR may promote individual gene transcription induced by GR; and it has been reported that vitamin D may upregulate the binding of GR to GRE [23]. These may be the biological mechanisms by which vitamin D enhances cellular responsiveness to glucocorticoids.

**Figure 2:**
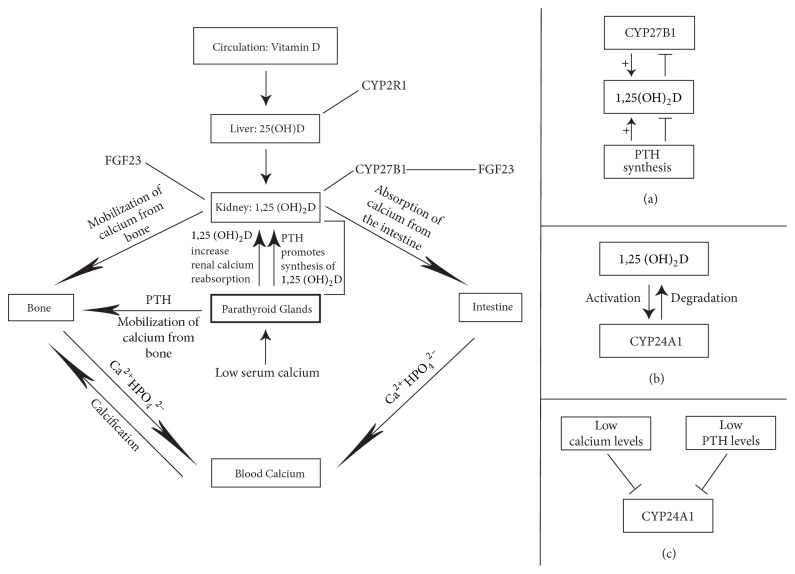
*Schematic representation of the functions of vitamin D in bone and calcium homeostasis.* Low serum calcium triggers the production and secretion of parathyroid hormone (PTH) by the parathyroid glands, which in turn induces the synthesis of 1,25(OH)_2_D in the kidney by CYP27B1. FGF 23 can inhibit renal tubular reabsorption of phosphate and CYP27B1 activity and can stimulate CYP24A1 [7, 8, 26, 27]. Vitamin D regulates calcium homeostasis by promotion of calcium absorption in the intestine, by reabsorption of calcium by the kidney, and by mobilization of calcium from the bone. 1,25(OH)_2_D through a negative feedback loop regulates the synthesis of PTH and of CYP27B1 (a) [4–6, 10]. Lower 25(OH)D is associated with increased PTH levels and lower bone density. This is owing to PTH/Vitamin D-induced increase in bone turnover, which results in increased bone resorption. Persistent vitamin D deficiency may result in osteopenia and osteomalacia [5]. 1,25(OH)_2_D promotes the production of CYP24A1, the enzyme that degrades 1,25(OH)_2_D (b) [1, 5], and low calcium and low PTH levels inhibit the production of CYP24A1 (c) [4]. Thus levels of circulating 1,25(OH)_2_D are regulated, in part, by CYP27B1-mediated production and by CYP24A1-mediated degradation [4].

The 1,25(OH)_2_D/VDR pathway, via transcriptional repression, downregulates the expression of the CYP27B1 (25(OH)D 1*α* hydroxylase) gene in the kidney, and the expression of the gene encoding parathyroid hormone (PTH), resulting in decreased production of 1,25(OH)_2_D [1, 4]. Thus, through a negative feedback loop, 1,25(OH)_2_D regulates both the synthesis of PTH and its own production (Figures 1 and 2).

## 3. 1,25(OH)_2_D/VDR in Calcium and Bone Homeostasis

In the intestine, the 1,25(OH)_2_D induced-calcium absorption is mediated by the epithelial calcium channel TRPV6 (transient potential vanilloid type 6) and in the kidney, 1,25(OH)_2_D mediates the active reabsorption of calcium via TRP5, in a mechanism similar to intestinal calcium absorption [4]. In the context of calcium homeostasis, the transcription of the gene encoding TRPV6 is regulated by vitamin D/VDR/RXR pathway [4].

Available information regarding the direct effect of vitamin D on bone is contentious. 1,25(OH)_2_D has the capacity directly or indirectly to regulate the proliferation, differentiation, and maturation of osteoblasts and osteoclasts, bone resorption, and mineralization [9]. It appears that 1,25(OH)_2_D via VDR upregulates the expression of genes encoding type 1 collagen, osteocalcin, and osteopontin that drive bone formation. In addition, 1,25(OH)_2_D/VDR induces the expression of RANK ligand by osteoblasts, which in turn mediates differentiation and increased activity of osteoclasts, thus ensuring bone turnover. This sequence of metabolic processes suggests that 1,25(OH)_2_D has the capacity to exert both anabolic and catabolic effects on bone [4, 5]. On a background of a low serum calcium level, regardless of the cause, 1,25(OH)_2_D/VDR-induced bone resorption is important in maintaining calcium homeostasis [4] (Figure 2).

However, not all studies have shown any significant correlation between levels of vitamin D and the bone turnover markers, bone-specific alkaline phosphatase, osteocalcin, procollagen type 1, N-terminal propeptide, and C-terminal telopeptide; and vitamin D supplementation has not been shown to have any effect on these markers in persons with low 25(OH)D, but with normal levels of calcium [9].

Thus, it appears that if the calcium balance is negative, 1,25(OH)_2_D/VDR directly mediates bone resorption with the mobilization of calcium from bone into the circulation, thus correcting the negative balance, but the effect of the 1,25(OH)_2_D/VDR on osteogenic cells in the face of a normal calcium balance is not fully understood. Under such circumstances in the context of normal calcium serum levels, 1,25(OH)_2_D regulates bone homeostasis indirectly through its effects on intestinal calcium absorption and renal calcium reabsorption, which maintain normal serum calcium levels [4].

Fibroblast growth factor 23 (FGF 23) is a protein primarily produced by osteocytes and osteoblasts that regulates the metabolism of vitamin D and the homeostasis of systemic phosphate. Once it is in the circulation, it can inhibit renal tubular reabsorption of phosphate, resulting in hypophosphatemia. Furthermore, by inhibiting CYP27B1 which converts 25(OH)D to 1,25(OH)_2_D in the kidney, and by stimulating CYP24A1 which catabolizes 25(OH)D and 1,25(OH)_2_D, FGF 23 suppresses the circulating 25(OH)D [7, 8, 26, 27]. Conversely, 1,25(OH)_2_D directly increases bone levels of FGF 23, probably via the upstream vitamin D response element in the FGF 23 gene promoter, in osteoblasts and osteocytes [26]. Thus, this bone-kidney feedback loop regulates production of FGF 23 in bone and of 1,25(OH)_2_D in the kidney, playing a role in the modulation of bone remodelling [27] (Figure 2).

1,25(OH)_2_D/VDR signalling in osteoblasts regulates expression of genes mediating differentiation of osteoblast precursor and bone mineralization, including the roles of alkaline phosphatase, osteocalcin, and osteopontin. Furthermore, 1,25(OH)_2_D/VDR signalling has the capacity to influence other signalling pathways involved in physiological activities of osteoblasts including transforming growth factor *β* (TGF-*β*), insulin growth factor 1 (IGF-1), bone morphogenic protein, interferon, PTH, hepatocyte growth factor (HGF), epidermal growth factor (EGF), and the Wnt/*β*-catenin signalling pathways [5, 28].

Osteoblasts express the CYP27B1 gene with the capacity to synthesize 1,25(OH)_2_D, but the biological significance of this is not clear. Once locally produced by osteoblasts, via a negative feedback loop, 1,25(OH)_2_D itself inhibits CYP27B1 expression, thus regulating its own production in bone (Figure 2); and it appears that interferon-*β* (INF-*β*) and interleukin-1 (IL-1) also play roles in the regulation of osteoblastic CYP27B1 [28].

The expression of specific osteogenic genes by osteoblasts in response to 1,25(OH)_2_D/VDR activation is dependent on the stage of differentiation of the cell and is influenced by immune cell-derived cytokines in the microenvironment [28].

However, as long as calcium levels are normal, bone metabolism is not affected by inhibition of 1,25(OH)_2_D/VDR signalling in osteoblasts. This is probably owing to the complex adaptive system which determines both osteoblast function and bone metabolism, in which deficiency of 1,25(OH)_2_D/VDR signalling is compensated by other factors in the system.

## 4. The Role of 1,25(OH)_2_D/VDR in Cancer

There is a strong biological rationale for the role of vitamin D deficiency in increasing cancer risk and for the use of vitamin D or its bioactive analogues for cancer prevention and treatment. VDR is expressed in most cancerous tissues; and results of* in vivo* animal studies and* in vitro* cell culture studies show that 1,25(OH)_2_D inhibits cell proliferation, angiogenesis, invasion and promotes differentiation and apoptosis. In cancer cells, 1,25(OH)_2_D/VDR activates cyclin-dependent kinase inhibitors (e.g., p21, p27), inhibits mitogenic growth factors such IGF-1 and EGF, and promotes the activity of TGF-*β*, thus inhibiting cell proliferation and cancer growth [4, 15].

1,25(OH)_2_D/VDR signalling has the capacity to downregulate cyclooxygenase-2, prostaglandin, and NF-kB pathways, thus inhibiting tumour-associated inflammation, to suppress antiapoptotic proteins (e.g., Bcl2) and to activate proapoptotic proteins (e.g., Bax, RAK). Acting together, all these can suppress cancer growth [4, 15].

The transcription factor NF-kB regulates the expression of genes controlling inflammation, cell proliferation, apoptosis, invasion, and metastasis [29]. It has been reported that 1,25(OH)_2_D/VDR can suppress NF-kB activities, resulting in decreased expression of proinflammatory cytokines and inhibition of cell proliferation and of cancer-associated inflammation, with slowing down cancer growth [1, 4].

Another potential anticancer mechanism of 1,25(OH)_2_D is through the interaction with the transforming growth factor-*β* (TGF-*β*) family of secreted proteins. Some of these proteins promote differentiation and apoptosis of epithelial cells but inhibit their proliferation. As outlined above, 1,25(OH)_2_D has the capacity to activate TGF-*β* signalling pathways, thus it may promote the tumour-suppressor activities [1].

The canonical Wnt signalling pathway is associated with translocation of *β*-catenin from adherence junctions to the nucleus with subsequent activation of the *β* catenin/T cell factor (TCF) transcriptional complex, and with the functional loss of E-cadherin. In cancer, activated Wnt/*β*-catenin signalling activates genes which promote increased proliferation, invasion, and metastasis of cancer cells [30]. The dysregulated Wnt/*β*-catenin signalling is observed early in carcinogenesis and is maintained throughout the course of cancer progression, but Wnt-associated activation of the epithelial-mesenchymal transition (EMT) cellular genetic program that results in cancer cell detachment, motility, and migration with subsequent invasion and metastasis is a relatively late event in tumourigenesis [30].

1,25(OH)_2_D/VDR enhances E-cadherin expression and nuclear export of *β*-catenin and induces the dickkopf 1, an extracellular inhibitor of Wnt signalling, all of which result in the inhibition of cancer growth [1, 4]. Furthermore, in cancerous keratinocytes, 1,25(OH)_2_D/VDR may alter the expression of genes encoding cytoskeletal proteins, affecting cytoskeletal organization and function resulting in increased cell adhesion and decreased cell motility, even further suppressing cancer growth [4].

Most cancers express not only the VDR but also CYP27B1 and CYP24A1 thus allowing cancer cells to regulate locally 1,25(OH)_2_D levels, and it has been reported that cancers expressing CYP27B1 tend to be well differentiated while cancers which do not express CYP27B1 tend to be poorly differentiated. Cancers which express CYP24A1 tend to be more aggressive than those which do not [4].

Evidence from* in vitro* studies support the concept of an antiproliferative effect of 1,25(OH)_2_D; but results of* in vivo* studies on laboratory animals show that the anticarcinogenic activity of 1,25(OH)_2_D mainly affects cancer progression rather than cancer initiation. In humans, the results of observational clinical studies of associations between vitamin D supplementation and cancer incidence do not show diminution in cancer incidence in response to vitamin D supplementation. However, one needs to be aware that most of these studies had objectives unrelated to cancer incidence and that while carcinogenesis is a slow biopathological process the duration of vitamin D supplementation and the follow-up period have been relatively short [4]. Differences in ethnicity, environmental living conditions, and life-style between populations in various geographic locations most likely influence the results of studies investigating the association between the level of circulating vitamin D and cancer risk [4].

## 5. Vitamin D in Relation to the Immune System

Many cells of both the innate and the adaptive immune systems express the VDR, and some of these cells also have the capacity to express the CYP27B1 and to produce the biologically active 1,25(OH)_2_D.

The molecular patterns of certain microorganisms activate the molecular pattern recognition receptor Toll-like receptor (TLR) 1/2 of innate immunocytes (e.g., monocytes, macrophages, and keratinocytes), resulting in upregulation of the expression of CYP27B1 and VDR, with the production of 1,25(OH)_2_D. In some of these cells 1,25(OH)_2_D/VDR signalling induces the expression of the genes encoding the antibacterial agents cathelicidin and *β*-defenisin. 1,25(OH)_2_D may also mediate antimicrobial effector responses independently of TLR receptors. Thus, it appears that vitamin 1,25(OH)_2_D/VDR signalling, in combination with, or independently of TLR signalling, fortifies the antibacterial responses of innate immune cells [17, 18, 31].

1,25(OH)_2_D can suppress the maturation of dedicated antigen presenting dendritic cells, resulting in reduction of their capacity to present antigens to naïve T lymphocytes in regional lymph nodes. This may lead to decreased antigen-specific T cell activation and proliferation, and possibly to T cell anergy [4, 18, 31].

It has been found that in laboratory animals 1,25(OH)_2_D/VDR signalling downregulates chronic T cell mediated hyperactive immunoinflammatory reactions through inhibition of T cell proliferation, and decreasing production of IL-2 and INF-*γ* by Th1 cells, and of IL17 by Th17 cells. 1,25(OH)_2_D/VDR-induced increase in the production of IL-4 by Th2 cells and in the activity of T regulatory cells (Treg) further moderates autoimmune T cell responses [4, 31, 32].

Despite these moieties of evidence derived from laboratory studies, in humans, the physiological significance of 1,25(OH)_2_D activity in the context of the immune system is not fully understood, and its role in the pathogenesis and in the clinical course of immunoinflammatory diseases is not clear. However, in general, as it is evident that in humans there is no causal association between vitamin D deficiency and the incidence or severity of immunoinflammatory diseases, augmenting treatment with vitamin D or its biologically active analogues does not improve the outcome of treatment [4, 17]. As medical science is not infallible, treatment practices that according to evidence-based experiments have not been found to be completely effective for the general population may still be successful for a subset of patients [33].

## 6. 1,25(OH)_2_D/VDR Signalling and the Cardiovascular System

VDR and CYP27B1 are observed in cardiac myocytes, cardiac fibroblasts, vascular smooth muscle, and endothelial cells, and it appears that activated 1,25(OH)_2_D/VDR pathways may well play some role in cardiovascular function [18].

Experimental studies in laboratory animals have shown that limitation of 1,25(OH)_2_D/VDR signalling can cause increased renin/angiotensin activity with hypertension and cardiac hypertrophy, reduction in the bioavailability of the vasodilator nitric oxide with consequent impaired blood vessel relaxation, endothelial cell dysfunction, upregulation of proinflammatory cytokines, and increased proliferation and migration of vascular smooth muscle cells [4, 34, 35].

In humans, results of observational studies on the association between low 25(OH)D and increased cardiovascular risk and of interventional clinical randomized controlled studies into the effects of supplementation of vitamin D or its bioactive analogues on cardiovascular risk have yielded inconsistent results. Therefore, while it is evident that 1,25(OH)_2_D/VDR signalling has the capacity to modulate several pathways involved in maintaining the homeostasis of endothelial cells and cardiac function, its biological significance under physiological conditions is not clear, and any role vitamin D deficiency may have in the pathogenesis of cardiovascular disease is not scientifically proven [4, 35].

## 7. Effects of Vitamin D on Skin Biology

The role of vitamin D as a regulator of skin physiology is complex. Vitamin D and its receptor are expressed by keratinocytes of the basal and spinous layers of the epithelium, and 1,25(OH)_2_D/VDR signalling influences proliferation, differentiation, and apoptosis of keratinocytes, and cutaneous immune responses [36].

In the basal cell layer of the epithelium, 1,25(OH)_2_D regulates proliferation of basal cell keratinocytes, while in the spinous cell layer it upregulates expression of agents of differentiation mediating the synthesis of keratins (K1, K10), involucrin, transglutaminase, loricrin, and filaggrin [36].* In vitro*, a high level of 1,25(OH)_2_D, via VDR-mediated genomic mechanisms, inhibits proliferation of keratinocytes [14] and via nongenomic mechanisms promotes differentiation of keratinocytes by increasing levels of intracellular calcium [36].

More mature keratinocytes in the upper layers of the epithelium produce proteins, lipids, and glucosylceramides which provide a physical epidermal barrier protecting the underlying tissue from penetration by infectious and toxic agents and also provide a permeability barrier [15, 20, 37].

Calcium signalling in keratinocytes is induced by the calcium sensing receptor (Casr), a plasma membrane-bound member of the G protein coupled receptor family, and cross-talk between the 1,25(OH)_2_D/VDR signalling and the Ca/Casr signalling pathways regulates the formation of plasma membrane E-cadherin/catenin complexes which form adherent junctions [37]. Synergism between Ca/Casr and 1,25(OH)_2_D/VDR signalling inhibits the translocation of *β*-catenin from the plasma membrane E-cadherin/catenin complex, thus diminishing the nuclear transcriptional activity of *β*-catenin [37].

Physiologically, the hedgehog intracellular signalling pathway regulates the differentiation and proliferation of keratinocyte stem cells. Functional upregulation of this pathway is an early genetic event in the pathogenesis of cutaneous basal cell carcinoma conferring upon affected basal keratinocytes enhanced proliferative capacity thus increasing risk of basal cell carcinoma. Furthermore, reduced functional activity of genes regulating repair of DNA damage caused by UVB radiation is also implicated in the pathogenesis of cutaneous basal cell carcinoma [38]. In this context as 1,25(OH)_2_D/VDR signalling has been shown to inhibit the hedgehog signalling pathway and to upregulate the activity of DNA nucleotide excision repair enzymes, 1,25(OH)_2_D has the potential to reduce the risk of basal cell carcinoma [14].

In the context of cutaneous squamous cell carcinoma, results of* in vitro* studies show that 1,25(OH)_2_D/VDR signalling inhibits cell proliferation by inducing cell cycle arrest, by triggering apoptosis, by downregulating DNA synthesis, and by promoting repair of UVB radiation-induced DNA damage [14, 20].

## 8. Vitamin D and Oral Health

It appears that 1,25(OH)_2_D/VDR plays a role in maintaining the homeostasis of oral epithelium and of oral immunity. VDR is expressed by oral keratinocytes in which it has a ligand-independent function in restraining proliferation of oral keratinocytes; and 1,25(OH)_2_D/VDR signalling has an even stronger inhibitory effect on proliferation of oral keratinocytes. Both* in vitro* and* in vivo* studies show that vitamin D deficiency brings about an increase in proliferation of oral keratinocytes, but without any morphological or histological alterations. Although it is clear that vitamin D deficiency in itself is not sufficient to cause precancerous transformation, synergistically with other genetic or environmental factors it may increase the risk of oral squamous carcinoma [39].

Since melanin diminishes the cutaneous production of vitamin D, black persons experienced vitamin D deficiency more frequently than white persons and this account in part for the higher prevalence of oral squamous cell carcinoma in black people than in white people [40].

The anti-inflammatory, antimicrobial, and immunomodulating effects of the 1,25(OH)_2_D/VDR pathway most probably play roles in maintaining the homeostasis of oral tissues in general, thus providing some protection against the development of bacterial plaque-induced periodontal diseases. There is evidence that vitamin D deficiency or VDR polymorphism are associated with increased risk of chronic periodontitis [41]. Thus supplementing the conventional treatment of chronic periodontitis with administration of biologically active vitamin D may have a beneficial effect [31, 42, 43].

## 9. Conclusion

1,25(OH)_2_D is regulated by a complex system of feedback mechanisms mediated by enzymes, hormones, and receptors. 1,25(OH)_2_D/VDR signalling has an essential role in calcium and bone homeostasis and has the capacity to regulate various cellular responses of the immune and cardiovascular systems, and to regulate differentiation, proliferation, and apoptosis of normal and malignant keratinocytes.

## Figures and Tables

**Table 1:** Causes of Vitamin D deficiency or functional impairment.

(i) Decreased exposure to sunlight
(ii) Impaired photosynthesis in the skin
(iii) Inadequate diet
(iv) Impaired intestinal absorption
(v) Impaired conversion of Vitamin D into active metabolites owing to liver or renal disease
(vi) Resistance to the biological effects of 1,25 (OH)_2_D
(vii) Accelerated inactivation of vitamin D metabolites (barbiturates, phenytoin, and rifampin)
(viii) Medications (anticonvulsants, systemic glucocorticoids, ketoconazole, or antiretrovirals)

## References

[1] Dimitrov V., Salehi-Tabar R., An B.-S., White J. H. (2014). Non-classical mechanisms of transcriptional regulation by the vitamin D receptor: Insights into calcium homeostasis, immune system regulation and cancer chemoprevention. *The Journal of Steroid Biochemistry and Molecular Biology*.

[2] Tang J. Y., Fu T., Lau C., Oh D. H., Bikle D. D., Asgari M. M. (2012). Vitamin D in cutaneous carcinogenesis. *Journal of the American Academy of Dermatology*.

[3] Searing D. A., Leung D. Y. M. (2010). Vitamin D in atopic dermatitis, asthma and allergic diseases. *Immunology and Allergy Clinics of North America*.

[4] Christakos S., Dhawan P., Verstuyf A., Verlinden L., Carmeliet G. (2016). Vitamin D: metabolism, molecular mechanism of action, and pleiotropic effects. *Physiological Reviews*.

[5] Bringhurst F. R., Demay M. B., Krane S. M., Kronenberg H. M., Kasper D. L., Fauci A. S., Hauser S. L., Longo D. L., Jameson L. J., Loscalzo J. (2015). Bone and mineral metabolism in health and disease. *Harrison's principles of internal medicine*.

[6] Kochwvar I. E., Taylor C. R., Krutmann J., Wolff K., Goldsmith L. A., Katz S. I. (2012). Fundamentals of cutaneous photobiology and photoimmunology. *in Fitzpatrick's Dermatology in General Medicine*.

[7] Holick M. F. (2007). Vitamin D deficiency. *The New England Journal of Medicine*.

[8] Hossein-Nezhad A., Holick M. F. (2013). Vitamin D for health: a global perspective. *Mayo Clinic Proceedings*.

[9] Schwetz V., Trummer C., Pandis M. (2017). Effects of vitamin D supplementation on bone turnover markers: A randomized controlled trial. *Nutrients*.

[10] Gallieni M., Cozzolino M., Fallabrino G., Pasho S., Olivi L., Brancaccio D. (2009). Vitamin D: Physiology and pathophysiology. *The International Journal of Artificial Organs*.

[11] Corcoran A., Nadkarni S., Yasuda K. (2016). Biological evaluation of double point modified analogues of 1,25-dihydroxyvitamin D2 as potential anti-leukemic agents. *International Journal of Molecular Sciences*.

[12] Holick M. F. (2004). Sunlight and vitamin D for bone health and prevention of autoimmune diseases, cancers, and cardiovascular disease. *American Journal of Clinical Nutrition*.

[13] Dawson-Hughes B., Heaney R. P., Holick M. F., Lips P., Meunier P. J., Vieth R. (2005). Estimates of optimal vitamin D status. *Osteoporosis International*.

[14] Tang J. Y., Fu T., Lau C., Oh D. H., Bikle D. D., Asgari M. M. (2012). Vitamin D in cutaneous carcinogenesis: Part II. *Journal of the American Academy of Dermatology*.

[15] Rosen C. J., Adams J. S., Bikle D. D. (2012). The nonskeletal effects of vitamin D: an endocrine society scientific statement. *Endocrine Reviews*.

[16] Rieger S., Zhao H., Martin P., Abe K., Lisse T. S. (2015). The role of nuclear hormone receptors in cutaneous wound repair. *Cell Biochemistry & Function*.

[17] Wei R., Christakos S. (2015). Mechanisms underlying the regulation of innate and adaptive immunity by vitamin D. *Nutrients*.

[18] Christakos S., Hewison M., Gardner D. G. (2013). Vitamin D: beyond bone. *Annals of the New York Academy of Sciences*.

[19] Xu Y., He B., Pan Y. (2014). Systematic review and meta-analysis on vitamin D receptor polymorphisms and cancer risk. *Tumor Biology*.

[20] Piotrowska A., Wierzbicka J., Zmijewski M. A. (2016). Vitamin D in the skin physiology and pathology. *Acta Biochimica Polonica*.

[21] Skversky A. L., Kumar J., Abramowitz M. K., Kaskel F. J., Melamed M. L. (2011). Association of glucocorticoid use and low 25-hydroxyvitamin D levels: results from the National Health and Nutrition Examination Survey (NHANES): 2001–2006. *The Journal of Clinical Endocrinology & Metabolism*.

[22] Hidalgo A. A., Trump D. L., Johnson C. S. (2010). Glucocorticoid regulation of the vitamin D receptor. *The Journal of Steroid Biochemistry and Molecular Biology*.

[23] Zhang Y., Leung D. Y. M., Goleva E. (2014). Anti-inflammatory and corticosteroid-enhancing actions of vitamin D in monocytes of patients with steroid-resistant and those with steroid-sensitive asthma. *The Journal of Allergy and Clinical Immunology*.

[24] Kalra N., Ishmael F. T. (2014). Cross-talk between vitamin D, estrogen and corticosteroids in glucocorticoid resistant asthma. *OA Inflammation*.

[25] Zhang Y., Leung D. Y. M., Goleva E. (2013). Vitamin D enhances glucocorticoid action in human monocytes: Involvement of granulocyte-macrophage colony-stimulating factor and mediator complex subunit 14. *The Journal of Biological Chemistry*.

[26] Wesseling-Perry K. (2010). FGF-23 in bone biology. *Pediatric Nephrology*.

[27] Mackenzie N. C. W., Zhu D., Milne E. M. (2012). Altered bone development and an increase in FGF-23 expression in Enpp1 -/- mice. *PLoS ONE*.

[28] van de Peppel J., van Leeuwen J. P. T. M. (2014). Vitamin D and gene networks in human osteoblasts. *Frontiers in Physiology*.

[29] Dekate K. (2014). Inflammation in the context of oral canc. *Oral Oncology*.

[30] Feller L., Kramer B., Lemmer J. (2012). Pathobiology of cancer metastasis: A short account. *Cancer Cell International*.

[31] Amano Y., Komiyama K., Makishima M. (2009). Vitamin D and periodontal disease.. *Journal of oral science*.

[32] Cantorna M. T., Snyder L., Lin Y.-D., Yang L. (2015). Vitamin D and 1,25(OH)_2_D regulation of T cells. *Nutrients*.

[33] Klein G., Brockman J. (2015). Evidence-based medicine. *This Idea Must Die*.

[34] Menezes A. R., Lamb M. C., Lavie C. J., DiNicolantonio J. J. (2014). Vitamin D and atherosclerosis. *Current Opinion in Cardiology*.

[35] Kienreich K., Grubler M., Tomaschitz A. (2013). arterial hypertension and cerebrovascular disease. *Indian J Med Res*.

[36] Barrea L., Savanelli M. C., Di Somma C. (2017). Vitamin D and its role in psoriasis: An overview of the dermatologist and nutritionist. *Reviews in Endocrine and Metabolic Disorders*.

[37] Bikle D. D., Jiang Y., Nguyen T., Oda Y., Tu C.-L. (2016). Disruption of vitamin D and calcium signaling in keratinocytes predisposes to skin cancer. *Frontiers in Physiology*.

[38] Feller L., Khammissa R. A. G., Kramer B., Altini M., Lemmer J. (2016). Basal cell carcinoma, squamous cell carcinoma and melanoma of the head and face. *Head & Face Medicine*.

[39] Yuan A., Woo S.-B. (2014). Adverse drug events in the oral cavity. *Oral Surgery, Oral Medicine, Oral Pathology, Oral Radiology, and Endodontology*.

[40] Lipworth L., Rossi M., McLaughlin J. K. (2009). Dietary vitamin D and cancers of the oral cavity and esophagus. *Annals of Oncology*.

[41] Dietrich T., Garcia R. I. (2005). Associations between periodontal disease and systemic disease: Evaluating the strength of the evidence. *Journal of Periodontology*.

[42] Miley D., Garcia M. N., Hildebolt C. F. (2009). Cross-Sectional study of vitamin d and calcium supplementation effects on chronic periodontitis. *Journal of Periodontology*.

[43] Bashutski J. D., Eber R. M., Kinney J. S. (2011). The impact of vitamin D status on periodontal surgery outcomes. *Journal of Dental Research*.

